# Predictors of suicidal behaviour in 36,304 individuals sickness absent due to stress-related mental disorders - a Swedish register linkage cohort study

**DOI:** 10.1186/1471-2458-13-492

**Published:** 2013-05-21

**Authors:** Kazi Ishtiak-Ahmed, Aleksander Perski, Ellenor Mittendorfer-Rutz

**Affiliations:** 1 Division of Insurance Medicine, Department of Clinical Neuroscience, Karolinska Institutet, Stockholm, Sweden; 2 Department of Public Health Sciences, Karolinska Institutet, Stockholm, Sweden; 3 Stockholm University, Stockholm Stress Centre, Stockholm, Sweden

**Keywords:** Suicide attempt, Suicide, Stress-related mental disorders, Sickness absence

## Abstract

**Background:**

Stress-related mental disorders (SRMD), which correspond to the diagnostic code F43 in the International Classification of Diseases, version 10, rank among the leading causes of sickness absence in several European countries. Despite the size of this health problem, research on risk factors for severe medical outcomes, like suicidal behavior, is lacking to date. The aim of this study was to investigate predictors of suicide attempt and suicide among sickness absentees with SRMD.

**Methods:**

A cohort of 36,304 non-retired individuals, aged 16–64 years on 31.12.2004, with at least one sickness absence spell due to SRMD, initiated in 2005, was followed up with regard to suicide attempt (2006–2009) and suicide (2006–2008). Univariate and multivariate hazard ratios (HR) with 95% confidence intervals (CI) were estimated for a number of predictors.

**Results:**

During the follow-up period, 266 individuals attempted suicide and 34 committed suicide. In the multivariate analyses, the following factors increased the risk of suicide attempt: =< 25 years of age, low educational level, lone parenthood, > 1 sickness absence spell, long duration of the first spell of sickness absence due to SRMD (> 180 days), > 4 and > 8 days of inpatient care due to somatic or mental diagnoses (2000–2005), and > 4 and > 1 outpatient visits due to somatic or mental diagnoses (2001–2005), respectively. Hazard ratios ranged from 1.4 to 4.2. Health care due to mental diagnoses and > 1 spell of sickness absence regardless of diagnosis were predictive of suicide.

**Conclusions:**

Several predictors related to socio-demographics, sickness absence and health-care consumption were identified as risk factors for suicidal behavior. Consideration of these risk factors is of both clinical and public health importance.

## Background

### Sickness absence due to stress-related mental disorders

Sickness absence due to mental disorders, particularly stress-related mental disorders, has increased in a number of European countries over the last 20 years, and currently comprises one of the major diagnostic groups among all cases of sickness absence [1,2]. Stress-related mental disorders correspond to diagnostic code F43 in the International Classification of Diseases (ICD, version 10), and include reactions to severe stress and adjustment disorders [3]. Despite the considerable increase and the size of the problem, research on the most serious medical outcomes, like the suicidal behaviour of sickness absentees with mental disorders and particularly stress-related mental disorders (SRMD) [4,5], is very limited [6,7]. Recent cohort studies have reported an increased risk of suicide in individuals on sick leave due to mental disorders in general [6], and on sick leave due to SRMD specifically [7].

Identifying risk individuals in time in order to prevent suicidal behavior is of utmost clinical and public health interest. Therefore, this study aimed to investigate the extent to which socio-demographic factors, as well as factors related to sickness absence and health-care consumption, predicted suicidal behavior in individuals sickness absent due to SRMD [8-11]. To the best of our knowledge, we are not aware of any previous study with a similar focus.

## Methods

The study was designed as a prospective cohort study by linking Swedish register data. All people 16 to 64 years-old on 31.12.2004 and registered in Sweden who initiated at least one sickness absence spell due to a stress-related mental disorder (SRMD) in 2005 (N= 38,870) comprised the study base. We excluded 2059 and 265 individuals who were on disability and old-age pension before 2005, respectively, and two further individuals who were erroneously coded as dead before 2005. Also, individuals with missing information on educational status (N=41), or on the length of the first sickness absence spell due to SRMD initiated in 2005 (N=201), were excluded. This left 36,304 individuals (26,315 women and 9,989 men) to be included in the study.

The following register data sources were used for all the individuals in the study: 1) Statistics Sweden: Longitudinal Integration Database for Health Insurance and Labour Market Studies (LISA), which includes socio-demographic information on age, sex, country of birth, place of residence, family situation and educational status; 2) the National Board of Health and Welfare: (i) the National Patient Register, which includes information on dates and diagnoses for inpatient and outpatient care, (ii) the Cause of Death Register, with data on dates and causes of death; 3) the Social Insurance Agency (SIA): Micro-Data for Analyses of Social Insurance (MiDAS), with information on date, grade and diagnosis of sickness absence and disability pension. The linkages were based on the unique personal numbers (de-identified) of all residents in Sweden.

### The Swedish social insurance system

All people above the age of 16, living in Sweden, with an income from work or unemployment benefit, who – due to disease or injury have a reduced work capacity – are covered by national sickness insurance and can receive sickness benefit [12]. After a first qualifying day, the employer is responsible for sick pay for the first 14 days of a sick leave spell; thereafter, sickness benefit is paid by the Social Insurance Agency. Self-employed people have more qualifying days, and they, as well as the unemployed, receive all sickness benefit from the Social Insurance Agency. A physician’s certificate is required after seven days of self-certification. For this study, data on sick leave with benefit from the Social Insurance Agency were employed.

### Diagnostic criteria

All diagnoses related to risk factors and outcomes were based on the corresponding codes of the International Classification of Diseases (ICD) in ICD 10. The definition of stress-related mental disorders in sickness absence spells was based on the corresponding ICD 10 Code F43.

### Measures of risk factors

Baseline socio-demographic characteristics from the LISA database were measured at the end of 2004 and categorized as shown in Table 1. Variables on sickness absence relate to spells initiated in 2005. Number of sickness absence spells regardless of diagnosis was categorised as follows: 1, 2 and > 2 spells. The variable on diagnosis-specific sickness absence spells was categorised as follows: only spells due to mental disorders, one additional spell due to a somatic disorder, and multiple additional spells due to somatic disorders. Duration of the first sickness absence spell due to SRMD initiated in 2005 was grouped in five categories: 1–14, 15–90, 91–180, 181–365, and more than 365 days. The following covariates were dichotomized: having at least one full-time period (at least one day with 100% sickness absence) in the first sickness absence spell due to SRMD; having at least one part-time period in the first sickness absence spell due to SRMD, and whether or not individuals had any repeated sickness absence spells due to SRMD.

**Table 1:** Descriptive statistics for individuals (N=36,304) sickness absent due to stress-related mental disorders

***Characteristics***	**N**	**%**
***Socio-demographic characteristics***		
Sex		
Female	26315	72.5
Male	9989	27.5
Age (years)		
17–25	1821	5.0
26–35	8216	22.6
36–45	11464	31.6
46–55	9138	25.2
56–65	5665	15.6
Education (years)		
0 to 9	4362	12.0
10 to 12	18066	49.8
Above 12	13876	38.2
Country of birth		
Sweden	31763	87.5
Other Nordic countries	1313	3.6
Other European countries	717	2.0
Outside Europe	2511	6.9
Place of residence		
Big cities	14017	38.6
Medium size cities	12869	35.4
Small town	9418	25.9
Family situation		
Married/cohabiting without children ^a^	4663	12.8
Married/cohabiting with children ^a^	13955	38.4
Single people without children ^a^	11490	31.6
Single people with children ^a^	6196	17.1
***Sickness absence characteristics ***^***b***^		
Repeated spells		
1 spell due to SRMD*	34569	95.2
> 1 spell due to SRMD*	1735	4.8
Spells with mental/somatic diagnoses		
Only spells with mental diagnoses	32586	89.8
One additional spell with somatic diagnoses	3330	9.2
Multiple spells with somatic diagnoses	388	1.1
Full-time ^c^		
No	4609	12.7
Yes	31695	87.3
Part-time ^d^		
No	18219	50.2
Yes	18085	49.8
Duration (days) ^e^		
1–14	10157	28.0
15–90	17370	47.8
91–180	3742	10.3
181–365	2469	6.8
> 365	2566	7.1
Number of spells ^f^		
1 spell	30048	82.8
2 spells	5291	14.6
> 2 spells	965	2.7
***Health care characteristics***		
Hospital stay due to mental diagnoses (days) ^g^		
No hospital stay	35036	96.5
1 to 8	663	1.8
> 8	605	1.7
Hospital stay due to somatic diagnoses (days) ^g^		
No hospital stay	25619	70.6
1 to 4	5797	16.0
> 4	4888	13.5
Outpatient care visits due to mental diagnoses ^g^		
No visits	33013	90.9
1	1763	4.9
> 1	1528	4.2
Outpatient care visits due to somatic diagnoses ^g^		
No visits	8806	24.3
1 to 4	16238	44.7
> 4	11260	31

^* ^SRMD – stress-related mental disorders; ^a ^Children living in the household; ^b ^Sickness absence due to stress-related mental disorders; ^c ^Individual had at least one full-time period (at least one day with 100% sickness absence) in the first sickness absence spell due to stress-related mental disorders; ^d ^Individual had at least one part-time period (day) in the first sickness absence spell due to stress-related mental disorders; ^e ^Duration of the first sickness absence spell due to stress-related mental disorders initiated in 2005; ^f ^Number of new sick leave spells initiated in 2005 regardless of diagnosis; ^g ^Cut-offs are based on the diagnosis-specific median.

Categorisation of health care variables was based on the diagnosis-specific median number of days and number of visits in inpatient (2000–2005) and outpatient care (2001–2005) (four variables). The median length of hospital stays due to mental and somatic diagnoses were 8 and 4 days, respectively, while the median numbers of visits in outpatient care due to mental and somatic diagnoses were one and four, respectively.

### Measurement of outcome

Individuals with inpatient care due to ICD-10 codes X60-X84 were regarded as cases of suicide attempt. We considered only the first suicide attempt during the follow-up period. People with similar ICD codes in the Cause of Death register were considered as cases of suicide.

### Statistical analysis

Cox proportional hazard regression models were applied after ensuring that the proportional hazard assumption was met. With regard to suicide attempt, individuals were followed from 01.01. 2006 to the date of inpatient care due to suicide attempt, death, emigration, or 31.12. 2009, whichever came first. With regard to suicide, follow-up was from 01.01. 2006 to the date of suicide, death by other causes than suicide, emigration, or 31.12. 2008, whichever came first. Hazard ratios for suicide attempt and suicide were estimated with 95% confidence intervals. The multivariate models included both significant predictors and significant confounders.

The partial likelihood ratio test was used to test for possible interactions between risk factors and to test the predictive value of variables in case of collinearity. Attributable proportions (APs) were estimated as follows: AP= (HR-1)/HR*f, where f denotes the proportion exposed to the various risk factors in all who attempted or committed suicide [13]. All statistical analyses were performed using SPSS version 20.

### Ethical considerations

The study population was based on several linked public national registers, derived from three different authorities. Ethical vetting is always required when using register data in Sweden. The vetting is performed by regional ethical review boards, and a risk appraisal associated with the Law on Public Disclosure and Secrecy is performed by data owners. The ethical review boards can, however, waive the requirement to consult the data subjects (or, in case of minors/children, next of kin, carers or guardians) directly to obtain their informed consent, and will often do so if the research is supported by an ethical review board and the data have already been collected in some other context. This project was evaluated and approved, according to these standards, by the Regional Ethical Review Board of Karolinska Institutet, Stockholm, Sweden.

## Results

In total, the 36,304 individuals contributed 143,836 person-years for suicide attempt and 108,520 person-years for suicide during the follow-up periods. The mean follow-up time was 3.96 years (standard deviation, SD: 0.33) for suicide attempt and 2.99 years (SD: 0.16) for suicide. With regard to suicide methods, out of the 34 suicides (0.1%), 17 (50%) were due to poisoning, and 12 (35%) to hanging. A majority (94%) of the 266 (0.7%) suicide attempts were due to poisoning.

Table 1 presents descriptive statistics on the study population and on the suicide attempt/suicide cases with regard to socio-demographic characteristics, sickness absence spells and health care. In terms of proportions, the study population was female (72.5%), aged between 36 and 45 years (31.6%), had 10 to 12 years of education (49.8%), was born in Sweden (87.5%), lived in bigger cities (38.6%), and was married/cohabiting with children living at home (38.4%). With regard to the sickness absence characteristics, most of the individuals had only one sickness absence spell due to SRMD initiated in 2005 (82.8%), had no additional sickness absence spells due to a somatic diagnosis (89.8%), had at least one full-time sickness absence period in the first sickness absence spell due to SRMD (87.3%), and 15–90 days of sickness absence due to SRMD (47.8%).

With regard to the health care characteristics, most of the individuals did not have any inpatient care due to either mental (96.5%) or somatic diagnoses (70.6%) between 2000 and 2005. At their first inpatient care stay due to a mental diagnosis in 2005, nearly half (43%) of the individuals had SRMD as a main diagnosis; 14% had a mental and behavioural disorder due to use of alcohol (ICD-10 code F10), and 16% a depressive episode (ICD-10 code F32) (data not shown).

The majority (90.9%) of the study population did not have any health care visits in specialized outpatient care due to a mental diagnosis (2001–2005). At the first outpatient care visit due to a mental disorder in 2005, the main diagnosis was a stress-related mental disorder in 50% of cases, while 16% and 13% of the individuals had a main diagnosis of a depressive episode and an anxiety disorder, respectively (data not shown). A considerable proportion of the individuals had made up to four health care visits (44.7%), or more than four health care visits (31%), due to somatic diagnoses (Table 1).

### Suicide attempt and risk factors

Most of the covariates were found to be significantly associated with suicide attempt in univariate analyses; the exceptions were sex, country of birth, and place of residence (Table 2). In the final model, young people (17–25 years-old) showed a two-fold increased risk of attempting suicide compared with people aged 36–45 years (Table 3). Age over 56 years was significantly protective. Further, low education (HR 2.43, 95% CI 1.63-3.63) and being single with children living at home HR 1.40 (95% CI 0.99–1.96) were predictive of suicide attempt (Table 3).

**Table 2:** Univariate hazard ratios (HR) and 95% confidence intervals (CI 95%) for suicide attempt in people sickness absent due to stress-related mental disorders, SRMD (N=36,304)

***Characteristics***	**N (%)**	**HR**	**CI 95%**
***Socio-demographic characteristics***			
Sex			
Male	65 (0.7)	0.86	0.65–1.13
Female	201 (0.8)	1	
Age (years)			
17–25	42 (2.3)	3.51	2.41–5.11
26–35	75 (0.9)	1.38	1.00–1.90
36–45	76 (0.7)	1	
46–55	60 (0.7)	0.99	0.71–1.39
56–65	13 (0.2)	0.35	0.19–0.63
Education (years)			
0 to 9	55 (1.3)	3.60	2.45–5.28
10 to 12	162 (0.9)	2.55	1.85–3.51
Above 12	49 (0.4)	1	
Country of birth			
Sweden	228 (0.7)	1	
Other Nordic countries	14 (1.1)	1.50	0.88–2.58
Other European countries	< 4 (0.3)	0.39	0.10–1.57
Outside Europe	22 (0.9)	1.23	0.79–1.90
Residence			
Big cities	105 (0.7)	1	
Medium sized cities	89 (0.7)	0.92	0.70–1.22
Small town	72 (0.8)	1.02	0.76–1.38
Family sitation			
Married/cohabiting with children ^a^	17 (0.4)	1	
Married/cohabiting without children ^a^	76 (0.5)	0.67	0.40–1.14
Single people without children ^a^	108 (0.9)	1.74	1.30–2.33
Single people with children ^a^	65 (1.0)	1.93	1.39–2.69
***Sickness absence characteristics ***^***b***^			
Repeated spells			
1 spell due to SRMD*	245 (0.7)	1	
> 1 spell due to SRMD*	21 (1.2)	1.71	1.10–2.70
Spells with mental/somatic disorders			
Only spells with mental disorders	221 (0.7)	1	
One additional spell with somatic disorders	38 (1.1)	1.70	1.20–2.40
Multiple spells with somatic disorders	7 (1.8)	2.70	1.27–5.71
Full-time ^c^			
No	13 (0.3)	1	
Yes	253 (0.8)	2.84	1.63–5.00
Part-time ^d^			
No	163 (0.9)	1	
Yes	103 (0.6)	0.63	0.5–0.81
Duration (days) ^e^			
1–14	48 (0.5)	1	
15–90	109 (0.6)	1.33	0.95–1.87
91–180	38 (1.0)	2.16	1.41–3.30
181–365	25 (1.0)	2.16	1.33–3.50
> 365	46 (1.8)	3.83	2.55–5.73
Number of spells (any disorders) ^f^			
1	184 (0.6)	1	
2	61 (1.2)	1.90	1.42–2.53
> 2	21 (2.2)	3.60	2.29–5.65
***Health care characteristics***			
Hospital stay due to mental disorders (days) ^g^			
No hospital stay	188 (0.5)	1	
1 to 8	34 (5.1)	9.89	6.90–14.25
> 8	44 (7.3)	14.25	10.26–19.79
Hospital stay due to somatic diagnoses (days) ^g^			
No hospital stay	116 (0.5)	1	
1 to 4	67 (1.2)	2.56	1.90–3.50
> 4	83 (1.7)	3.79	2.86–5.03
Outpatient care visits due to mental disorders ^g^			
No visits	161 (0.5)	1	
1	33 (1.9)	3.88	2.67–5.64
> 1	72 (4.7)	10.0	7.54–13.15
Outpatient care visits due to somatic disorders ^g^			
No visits	23 (0.3)	1	
1 to 4	79 (0.5)	1.87	1.17–2.97
> 4	164 (1.5)	5.63	3.64–8.71

^* ^SRMD – stress-related mental disorders, ^a ^Children living at home; ^b ^Sickness absence due to stress-related mental disorders; ^c ^Individual had at least one full-time period (at least one day with 100% sickness absence) in the first sickness absence spell due to stress-related mental disorders; ^d ^Individual had at least one part-time period (day) in the first sickness absence spell due to stress-related mental disorders; ^e ^Duration of the first sickness absence spell due to stress-related mental disorders initiated in 2005; ^f ^Number of new sick leave spells initiated in 2005 regardless of diagnosis; ^g ^Cut-offs are based on the diagnosis-specific median.

**Table 3:** Multivariate adjusted hazard ratios (HR) and 95% confidence intervals (CI 95%) for suicide attempt in people sickness absent due to stress-related mental disorders (N=36,304)

***Characteristics***	HR*	**CI 95%**
***Socio-demographic characteristics***		
Age (years)		
17–25	1.98	1.31–2.98
26–35	1.18	0.86–1.64
36–45	1	
46–55	1.13	0.80–1.60
56–65	0.44	0.24–0.82
Education (years)		
0 to 9	2.43	1.63–3.63
10 to 12	2.00	1.45–2.78
Above 12	1	
Family situation		
Married/cohabiting with children ^a^	1	
Married/cohabiting without children ^a^	0.95	0.54–1.66
Single people without children ^a^	1.23	0.89–1.7
Single people with children ^a^	1.40	0.99–1.96
***Sickness absence characteristics ***^***b***^		
Duration (days) ^c^		
1–14	1	
15–90	1.19	0.85–1.68
91–180	1.85	1.20–2.84
181–365	1.86	1.14–3.05
> 365	2.20	1.44–3.35
Number of spells (any disorders) ^d^		
1	1	
2	1.44	1.07–1.94
> 2	2.02	1.27–3.20
***Health care characteristics***		
Hospital stay due to mental diagnoses (days) ^e^		
No hospital stay	1	
1 to 8	3.37	2.25–5.03
> 8	4.15	2.84–6.06
Hospital stay due to somatic diagnoses (days) ^e^		
No hospital stay	1	
1 to 4	1.51	1.10–2.06
> 4	1.86	1.36–2.53
Outpatient care visits due to mental diagnoses (days) ^e^		
No visits	1	
1	1.85	1.25–2.74
>1	3.08	2.20–4.31
Outpatient care visits due to somatic diagnoses (days) ^e^		
No visits	1	
1–4	1.36	0.85–2.18
> 4	2.41	1.51–3.85

^* ^Analyses are adjusted for sex, age, education, type of family, duration of first sickness absence spell due to SRMD, number of spells due to SRMD, and all health care related predictors, ^a ^Children living at home; ^b^ Sickness absence due to stress-related mental disorders; ^c^ Duration of the first sickness absence spell due to stress-related mental disorders initiated in 2005; ^d ^Number of new sick leave spells initiated in 2005 regardless of diagnosis; ^e ^Cut-offs are based on the diagnosis-specific median.

In the multivariate model, people who had taken more than 365 days of sick leave due to SRMD during the first spell initiated in 2005 showed more than twice the risk of suicide attempt of people who had only 1–14 days of sickness absence (Table 3). The related attributable proportion (AP) was 3.15%. Individuals with more than two sickness absence spells initiated in 2005, regardless of diagnosis, also showed a two-fold increased risk of suicide attempt compared with individuals with only one such spell (HR 2.02, 95% CI 1.27-3.20; AP 6.40). All the other variables related to sickness absence spells were only significant in the univariate models.

The adjusted HR and AP were 4.15 (95% CI 2.84–6.06) and 4.59, respectively, for individuals who had more than eight hospital days due to mental diagnoses during the years 2000 through 2005, compared with individuals who did not have any hospital stay (Table 3). The adjusted HR was increased three-fold, and the AP was 2.49%, for people who had made more than one outpatient health care visit during the years 2001 through 2005 due to a mental diagnosis compared with individuals who did not make any such health care visits. Both inpatient and outpatient health care due to somatic diagnoses increased the suicide-attempt risk two-fold in the multivariate analyses (APs of 1.48% and 0.95%, respectively).

### Suicide and risk factors

Male gender, young age, being single without children living at home, having two sick leave spells regardless of diagnosis and diagnosis-specific inpatient and outpatient health care were found to be significantly associated with suicide in the univariate analyses (Table 4). In the multivariate model, having two sick leave spells, regardless of diagnosis, increased the risk of committing suicide more than two-fold compared with having only one spell (HR 2.20, 95% CI 1.07–4.55) (Table 5). The related AP was 1.69%. People with previous inpatient care due to a mental diagnosis exceeding eight days had a three-fold increased risk of committing suicide compared with individuals without mental health care in the multivariate model (AP 4.80) (Table 5). The adjusted HRs for one and more than one outpatient care visit due to mental disorders were 3.69 (95% CI 1.30–10.50) and 7.89 (95% CI 3.34–18.67), respectively (Table 5); the APs were 5.02 and 2.29, respectively.

**Table 4:** Univariate hazard ratios (HR) and 95% confidence intervals (CI 95%) for suicide in people sickness absent due to stress-related mental disorders (N=36,304)

**Characteristics**	**N (%)**	**HR**	**CI, 95%**
***Socio-demographic characteristics***			
Sex			
Male	16 (0.16)	2.35	1.20–4.61
Female	18 (0.07)	1	
Age (years)			
17–25	6 (0.33)	3.44	1.27–9.29
26–35	4 (0.05)	0.51	0.16–1.59
36–45	11 (0.10)	1	
46–55	12 (0.13)	1.37	0.60–3.10
56–65	< 4 (0.02)	0.19	0.02–1.43
Education (years)			
0 – 9	< 4 (0.07)	1.45	0.43–4.91
10 – 12	18 (0.10)	1.36	0.39–4.78
> 12	13 (0.09)	1	
Country of birth			
Sweden	29 (0.09)	1	
Other Nordic countries	0 (0.00)	-	-
Other European countries	0 (0.00)	-	-
Outside Europe	5 (0.20)	2.19	0.85–5.65
Place of residence			
Big cities	14 (0.10)	1	
Medium size cities	12 (0.09)	0.93	0.43–2.02
Small town	8 (0.08)	0.85	0.36–2.03
Family sitation			
Married/cohabiting with children ^a^	< 4 (0.02)	1	
Married/cohabiting without children ^a^	8 (0.06)	0.38	0.05–3.00
Single people without children ^a^	19 (0.17)	2.90	1.27–6.62
Single people with children ^a^	6 (0.10)	1.69	0.59–4.87
***Sickness absence characteristics ***^***b***^			
Repeated spells			
1 spell due to SRMD*	32 (0.09)	1	
> 1 spell due to SRMD*	< 4 (0.12)	1.25	0.30–5.19
Spell with mental/somatic disorders			
Only spells with mental disorders	30 (0.09)	1	
One additional spell with somatic disorders	4 (0.12)	1.31	0.46–3.72
Multiple spells with somatic disorders	0 (0.00)	-	-
Full-time ^c^			
No	0 (0.00)	-	-
Yes	34 (0.11)	-	-
Part-time ^d^			
No	21 (0.12)	0.62	0.31–1.24
Yes	13 (0.07)	1	
Duration (days) ^e^			
1–14	7 (0.07)	1	
15–90	17 (0.10)	1.42	0.59–3.43
91–180	4 (0.11)	1.56	0.45–5.30
181–365	5 (0.20)	2.95	0.94–9.30
> 365	< 4 (0.04)	0.57	0.07–4.60
Number of spells (any disorders) ^f^			
1	23 (0.08)	1	
2	11 (0.21)	2.73	1.33–5.59
> 2	0 (0.00)	-	-
***Health care characteristics***			
Hospital stay due to mental disorders (days) ^g^			
No hospital stay	23 (0.07)	1	
	5 (0.75)	11.61	4.41–30.53
> 8	6 (0.99)	15.23	6.20–37.41
Hospital stay due to somatic disorders (days) ^g^			
No hospital stay	20 (0.08)	1	
1 to 4	5 (0.09)	1.11	0.42–2.95
> 4	9 (0.18)	2.37	1.08–5.20
Outpatient care visits due to mental disorders ^g^			
No visits	16 (0.05)	1	
1	5 (0.28)	5.87	2.15–16.02
> 1	13 (0.85)	17.68	8.51–36.76
Outpatient care visits due to somatic disorders ^g^			
No visits	< 4 (0.02)	1	
1 to 4	15 (0.09)	4.07	0.93–17.80
> 4	17 (0.15)	6.66	1.54–28.83

^* ^SRMD – stress-related mental disorders, ^a ^Children living at home; ^b ^Sickness absence due to stress-related mental diagnoses; ^c ^Individual had at least one full-time period (day) in the first sickness absence spell due to stress-related mental disorders; ^d^ Individual had at least one part-time period (day) in the first sickness absence spell due to stress-related mental disorders; ^e ^Duration of the first sickness absence spell due to stress-related mental disorders initiated in 2005; ^f ^Number of new sick leave spells initiated in 2005 regardless of disorders; ^g ^Cut-offs are based on the diagnosis-specific median.

**Table 5:** Multivariate Hazard ratios (HR) and 95% Confidence Intervals (CI 95%) for suicide in people sickness absent due to stress-related mental disorders (N=36,304)

**Characteristic**	HR *	**CI, 95%**
***Sickness absence characteristics ***^***a***^		
Number of spells (any disorders) ^b^		
1	1	
2	2.20	1.07–4.55
***Health care characteristics***		
Hospital stay due to mental disorders (days) ^c^		
No hospital stay	1	
1 to 8	3.47	1.22–9.92
> 8	3.66	1.33–10.07
Outpatient care visits due to mental diagnoses ^c^		
No visits	1	
1	3.69	1.30–10.50
> 1	7.89	3.34–18.66

^* ^HR adjusted for sex, age, family situation, number of sick spells (any disorders) and days of hospital stay and outpatient care visits due to mental diagnoses; ^a ^Sickness absence due to stress-related mental diagnoses; ^b ^Number of new sick leave spells initiated in 2005 regardless of diagnosis; ^c ^Cut-offs are based on the diagnosis-specific median.

## Discussion

Young age, low education, lone parenthood, increasing numbers of days and spells of sickness absence, and inpatient and outpatient care due to mental and somatic diagnoses were predictive of suicide attempt in individuals who were sickness absent due to stress-related mental disorders. Previous and on-going inpatient and outpatient health care due to mental diagnoses and having two compared with only one spell of sickness absence predicted completed suicide.

### Methodology

To the best of our knowledge, this study is the first to investigate predictors of suicide attempt and suicide among individuals with sickness absence due to stress-related mental disorders. The main strength of the study lies in the large data set that covers the whole Swedish population using register data of good quality [14-16]. Another strength is the prospective study design that includes four and three years of follow-up for suicide attempt and suicide, respectively, with practically no dropout. The study was also able to analyse a wide variety of risk factors related to socio-demographics, characteristics of sickness absence spells, and diagnosis-specific health care.

### Limitations

The limitations of the study should also be mentioned, which include the limited power of the study with regard to the multivariate analysis of suicide as outcome. Another issue is the validity of sickness absence diagnoses, which is often discussed. A previous Swedish study, however, judged the validity of sickness absence diagnoses to be good [15]. In addition there is an on-going debate regarding whether and how stress-related mental disorders can be distinguished from depression; for example, post-traumatic stress disorders and depression often co-occur, and adjustment disorders in particular are difficult to distinguish from major depression [17]. Nevertheless, individuals sickness absent due to stress-related mental disorders differed considerably from the individuals absent due to depression found in a previous study, particularly with regard to suicide risk and degree of health-care consumption [7]. In addition, guidelines related to the diagnosis of stress-related mental disorders from the National Board of Health and Welfare in Sweden indicate clearly that if another disorder, e.g. depression, is present, depression should be used as the main diagnosis [18].

Since only the main diagnosis is recorded for any sickness absence spell in the Social Insurance Agency’s register, we have missed individuals with a stress-related mental disorder as a secondary diagnosis. Also, we could not adjust for other mental and somatic diagnoses as comorbidities involved in the cases of sickness absence. Comorbid diagnoses are established predictors of suicidal behaviour [19]. Nevertheless, we could analyse the effect of inpatient and outpatient care due to a somatic or mental diagnosis as a measure of morbidity.

In this study, we only considered cases of suicide attempt recorded in inpatient care. A study in Sweden has reported that approximately 47% of suicide attempters in the general population do not seek medical care after their suicide attempt [20]. We therefore expect that we only captured suicide attempts of greater medical severity.

### Socio-demographic factors

We found that young age, low education and living alone with children were associated with an increased risk of suicide attempt, which is consistent with the findings of previous studies of the general population and of patients with mental disorders in other treatment settings [21]. Young people, in particular, are known to react to stressors and adverse life events more impulsively with suicidal behavior than people who are older [22] Awareness of these risk factors seems crucial to preventing suicide attempts in this patient group.

### Sickness absence characteristics

The risk of attempting suicide was found to increase with increasing duration of sick leave and number of spells. Also, having 2 spells of sick leave, regardless of diagnosis, increased the risk of suicide more than two-fold. We have not been able to find any previous studies analysing the relation between frequency and duration of sickness absence spells and the risk of suicide attempt or suicide in individuals sickness absent due to stress-related mental disorders. Our findings are consistent with previous reports showing a 6-fold increased risk of suicide if there is more than one sick leave spell due to a psychiatric diagnosis after adjusting for age, gender, occupational grade, and sickness absence due to other diagnoses [6]. In addition, several studies have found sickness absence and increasing number of sickness absence days to be associated with suicide [23,24]. Our study is the first to show that sickness absence continues to be a risk factor even after controlling for inpatient and outpatient health care due to mental and somatic diagnoses.

Duration and number of spells of sick leave may not solely be measures of the recurrence, severity and chronicity of the disorder [25] underlying sickness absence, but might also reflect inadequacy of treatment and rehabilitation efforts [25]. Furthermore, increasing length of sickness absence might be associated with delayed help-seeking, and adverse health behaviours like alcohol consumption and social isolation [26], which in turn can increase the suicide risk [27,28]. Early rehabilitation, measures to improve the working environment and facilitate return-to-work, and also adequate treatment and follow-up of patients seem warranted in order to prevent suicidal behaviour. These findings have strong implications for primary health care, since the majority of cases of sickness absence are managed in primary health care [7].

### Inpatient and outpatient health care

Long hospital stays and frequent outpatient care visits due to mental diagnoses were found to be strongly associated with suicidal behaviour among individuals on sick leave due to SRMD. Although length of inpatient care due to mental diagnoses per se has been linked to an increased suicide risk [10,11,29-31], we are not aware of any previous studies investigating the effect of diagnosis-specific health care on patients on sick leave due to SRMD. Our results show an increased risk of suicide attempt with increasing days and visits to inpatient and outpatient care due to somatic diagnoses. Possible explanations for these findings include the association of somatic disorders with suicidal behaviour, and also potentially under-diagnosed mental disorders in individuals seeking health care due to somatic complaints [19,32]. Several mechanisms are likely to link somatic disorders to suicidal behaviour, e.g. the pain associated with the somatic disorder, or the psychological strain associated with a lethal somatic diagnosis such as cancer [19,32]. Adequate treatment and thorough follow-up of patients on sick leave due to SRMD with a history of frequent health care contacts due to both mental and somatic disorders are therefore warranted for the purpose of preventing suicidal behaviour.

## Conclusions

Young age, low education, lone parenthood, long and frequent sickness absence spells and inpatient and outpatient care due to somatic and mental diagnoses are predictive of suicide attempt in individuals sickness absent due to stress-related mental disorders. With regard to suicide completion, frequent sickness absence spells, and also inpatient and outpatient care due to mental disorders turned out to be risk factors. It is of public health and clinical importance to be aware of these risk factors when designing strategies to prevent suicidal behavior in individuals on sick leave due to stress-related mental disorders. These findings have particular importance in primary health care settings, where the vast majority of sickness certification due to stress-related mental disorders takes place.

## Competing interests

The authors have no competing interests to declare.

## Authors’ contributions

EMR is responsible for the core idea and study design. KIA and EMR carried out the data analyses and drafted the manuscripts. EMR, KIA and AP contributed to successive drafts and agreed on the final version of the manuscript. All authors read and approved the final manuscript.

## Pre-publication history

The pre-publication history for this paper can be accessed here:

http://www.biomedcentral.com/1471-2458/13/492/prepub
